# A Newly Reported Late Complication of Endoscopic Fundoplication: A Case Report

**DOI:** 10.3390/reports7010012

**Published:** 2024-02-09

**Authors:** Walid Bukhari, Hager Aref, Mohammed Ghunaim, Ahmed Elaryan

**Affiliations:** 1 International Medical Center, Jeddah 23324, Saudi Arabia; 2 Faculty of Medicine, King Abdulaziz University, Jeddah 21589, Saudi Arabia

**Keywords:** late complication, endoscopic fundoplication, laparoscopic technique, gastro-gastric fistula, gastroesophageal reflux disease

## Abstract

Endoscopic methods are emerging as a possible adjunct to surgery in the management of gastroesophageal reflux disease. Initially, endoscopic treatment has failed because of inefficient suturing devices, and although over many years it proved to be safe, it still can result in serious adverse events. In this article, the authors present a rare case of a male patient who experienced severe reflux years following endoscopic fundoplication. Moreover, in this report, we discovered an interesting finding with the patient who was diagnosed and managed laparoscopically. Although considered minimally invasive, endoscopic fundoplication can have significant deleterious consequences, and early recognition of these complications is vital to limit associated morbidities.

## 1. Introduction

Gastroesophageal reflux disease (GERD) is a condition that affects a significant number of people worldwide. It is marked by symptoms, such as heartburn, regurgitation, and dyspepsia, as a result of the reflux of the stomach acid content into the esophagus [1]. Over the years, the conventional approach to the management of GERD has basically relied on lifestyle modifications, medications, and surgical procedures if needed [2]. The endoscopic treatment of Gastroesophageal Reflux Disease (GERD) has emerged as a possible alternative to surgical fundoplication. In the early period of the introduction of this technique, there were concerns regarding the efficacy and complications related to this novel method [1,2,3].

Over the past years, it has demonstrated acceptable safety and a reasonable efficacy profile—about 90% long-term effectiveness for the treatment of GERD [4,5]. Recent studies have revealed promising results in the management of GERD using endoscopic therapy such as transoral incisionless fundoplication (TIF) and radiofrequency ablation (RFA) [6]. However, long-term success is yet to be validated through well-designed randomized controlled trials (RCTs) [6,7]. The complex nature of GERD, its multifactorial pathophysiology, and the transient anatomical changes caused by endoscopic procedures have limited its success [7]. To date, the procedure has demonstrated an improvement in GERD symptoms and cessation or reduction in proton pump inhibitor usage in about 75% of cases [8].

However, a recent approach used endoscopic fundoplication (EF), which is carried out by the use of suturing devices to create a fold in the stomach to prevent reflux. The suturing is carried out to wrap the lower esophageal sphincter. Earlier attempts at the use of EF were hindered by the efficiency of the suturing devices. Despite the challenges, advancements in the use of technology and procedural techniques have made it a safe and more feasible alternative to managing GERD, hence transforming EF [4]. Endoscopic fundoplication is regarded as a minimally invasive procedure used to manage GERD and its symptoms. It is performed by forming a partial barrier between the stomach and the esophagus to prevent acid reflux into the esophagus [4]. The American Gastroenterological Association (AGA) recommends it for “Patients with esophagitis who are intolerant of PPI therapy, Patients with symptoms of the esophageal GERD syndrome poorly controlled by PPI therapy, especially in the setting of persistent troublesome regurgitation, carefully selected patients with extraesophageal GERD syndromes in whom a reflux causality has been established to the greatest degree possible” as the indication for possible endoscopic fundoplication [9].

Although they are considered minimally invasive, endoscopic procedures for GERD treatment can lead to deleterious consequences, and early recognition of these adverse events is a crucial step to decrease associated morbidity and mortality [10]. These complications may include belching difficulty, dysphagia, hiatus disruption, and strictures, which may affect the quality of the patient’s life and, in severe cases, may require surgery to correct or dismantle the fundoplication. There were also reports of GERD laparoscopic surgical cases associated with gastric necrosis and gastropericardial fistulas. This has triggered debate among the medical community regarding the appropriate application of this method in managing GERD [5].

However, it is pertinent to identify rare complications, which may arise as either adverse effects of treatment or procedures that were previously underreported, to improve patient care and generate useful clinical findings [11]. EF is recommended for patients who are experiencing GERD symptoms with mild to moderate anatomical abnormalities in the gastroesophageal junction without large hiatal hernias or esophageal motility problems [12]. However, hiatal hernias that are larger than 2 cm are treated with endoscopic TIF procedures and may require cTIF to repair the hiatal hernia [13].

This report contains a rare incidence of a male patient who, despite the effectiveness and safety of this procedure, presents with severe reflux years after EF. The case report also sheds more light on laparoscopic diagnosis and management of this late complication, emphasizing the understanding and early identification of EF-related adverse events and the importance of recognizing and addressing the consequences associated with this procedure.

In this paper, the authors are the first to report this case in the literature, a case of a young male patient who experienced severe reflux and intolerance to oral intake secondary to a gastro-gastric fistula after treatment for GERD with EF.

## 2. Detailed Case Description

### 2.1. Pre-Procedure

**Figure 1:**
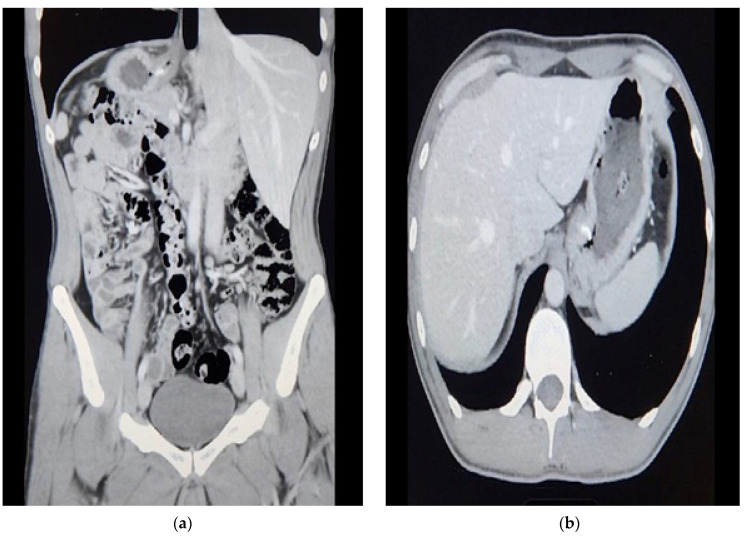
CT scan of the abdomen pelvis: (**a**) Coronal; (**b**) Transverse. Suspicious increased wall thickening of the proximal stomach extending to the gastroesophageal junction.

This is a case of a 31-year-old male patient from Saudi Arabia who was otherwise medically free and presented with status post-endoscopic fundoplication that was performed in another country 3 years ago. The patient reported that he developed a post-operative hemothorax that was managed immediately with a right thoracotomy and chest tube. He came to the clinic with a complaint of severe gastroesophageal reflux. As per the patient, it was worse than the first operation. We referred him to a gastroenterologist for his opinion, and he believed the first operation failure could predict the second trial failure, so a surgical approach was preferable. He had a follow-up upper GI endoscopy performed at another hospital, of which no report is available to us. A CT scan was performed with both oral and IV contrast in one of the hospitals; images reviewed in our hospital showed no chest pathology, yet the abdomen showed a hiatal hernia and stomach thickening, describing the ancient fundoplication folds (Figure 1). Esophagogastroduodenoscopy (EGD) was carried out, and it revealed normal findings.

The patient was admitted on 31 January 2023 for elective Laparoscopic revision of the Nissen fundoplication and hiatal hernia (HH) repair. During the operation, a gastric fistula was discovered and was resected; a large hiatal hernia was identified. It was reduced and repaired, and fundoplication was performed.

### 2.2. Operation Note

Laparoscopic Nissen fundoplication: redo, hiatal hernia repair, excision of gastro-gastric fistula: 31 January 2023.

**Figure 2:**
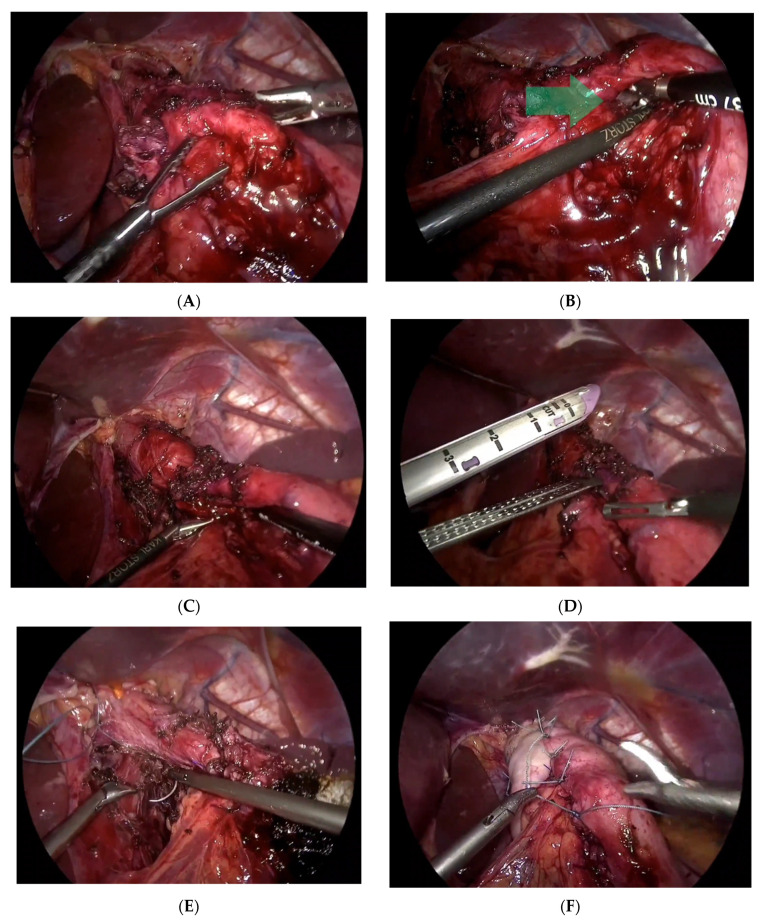
Laparoscopic procedures in the management of endoscopic fundoplication complications (**A**) exploring the previous endoscopic fundoplication; (**B**) a deep, full-thickness endoscopic stitch and fistula (the fistula is pointed by the green arrow); (**C**) using a nasogastric tube to confirm the presence of a fistula; (**D**) cutting the fistula using GIA; (**E**) repair of the hiatus; (**F**) Nissen fundoplication ended by Rossetti stitch.

Under general anesthesia in the supine open-leg position, cleaning and toweling were performed. Three trocars were inserted at 11, 12, and 5 mm after insufflation of the abdomen by CO_2_ up to 14 mmHg pressure. A 2 mm liver retractor was used as it was a huge fatty liver. On examination, there was a gastro-gastric fistula, in addition to a hiatal hernia, which was easily reduced from the mediastinum after adhesiolysis (Figure 2). And there was a complete exposure of the esophageal hiatus.

The gastrohepatic ligament was opened over the caudad lobe of the liver using the ligasure device, and the hepatic branch of the vagus nerve was identified and carefully preserved. The peritoneal incisions were extended over the left and right crus, and the mediastinum was entered. The esophagus was circumferentially mobilized, and all vessels encountered were controlled by the ligasure. The short gastric vessels were divided to the level of the left crus to ensure that the entire posterior aspect of the upper fundus of the stomach was completely mobilized. Upon careful dissection and release of the old endoscopic Nissen stitches, we identified a gastro-gastric fistula. The fistula was confirmed by the passage of a nasogastric tube of large caliber. Dissection and excision of the fistula tract were performed using GIA purple. A stabler of size 60 mm twice, followed by oversuturing the stabler lines by PDS 3-0.

We confirmed that there are no gastric injuries. Proceeding to the closure of the diaphragmatic defect, we closed it posteriorly using Ethibond 2-0. Then, the fundoplication was completed using interrupted 2-0 Ethibond sutures (crurogastric, cranoesophageal, and gastrogastric) and modified toupet stitches to fix the wrap and avoid intrathoracic slippage. The suture line was oriented at the 10 to 11 o’clock position, and the superior and inferior sutures were anchored to the wall of the esophagus. The medial stitch was placed between the walls of the fundus of the stomach. The calibration tube was easily removed and reinserted twice to make sure the passage was patent and there was no twist or narrowing in the stomach or evidence of tension.

Hemostasis was maintained, and snow was used at the stitches line, yet removed before the end of the operation. A lymph node was removed during dissection and sent for histopathology testing. In the end, the fascia right trocar and umbilical trocar sites were closed by a suture passer of Vicryl 0. This was followed by Vicryl 3/0, then Monocryl 3-0. Dressing was applied.

The patient was then awakened and transported back to the recovery room in satisfactory condition, with sponge and needle counts reported as correct at the end of the procedure.

### 2.3. Post Procedure

Post-laparoscopic Nissen fundoplication, HH repair and the excision of the gastric fistula were seen by the team on day 2. The patient complained of mild epigastric pain, no vomiting, and no gases yet; he was given simethicone. The abdomen was soft and lax with mild distention, bloated by gases, and had tender surgical sites. This was mobilized the following day using a spirometer. Laboratory results revealed that CBC showed Hb at 13.1 g/dL; WBC 8 (normal was 10).

Barium swallow showed a dilatation of the esophagus with evidence of breaking at the gastroesophageal junction. There were no filling defects or masses in the esophagus. The contrast passes slowly to the stomach, which appears unremarkable. There was no reflux in the delayed images. The mucosa appears smooth, with no thickening or ulceration. There is no evidence of diverticulae, tertiary contrast shoulder, or dysmotility. The impression was that the thickening was describing the fundoplication wrap. Given the anti-inflammatory protocol to reduce edema, we kept him NPO.

On 2 February 2023, the patient felt better and was able to drink fluids with no problems, no dysphagia, no reflux, and no vomiting. During the post-operative observation, he recovered smoothly and tolerated oral intake, and the workup showed stable hemoglobin and a normal white blood count. In addition, a barium swallow demonstrated no filling defect, no reflux, and that the contrast was passing to the stomach nicely. On the second day following the operation, he tolerated oral intake, reported no reflux, and passed gases. Furthermore, he was pain-free and mobilized; as a result, he was ready to go home after fulfilling the discharge criteria. The patient was ready to be discharged home, fit the criteria of discharge, passed gases, and tolerated oral intake.

He was placed on the plan to be discharged home, with OPD follow-up carried out in one week, and continue on PPI and antiemetics.

In the week following the operation, he reported to the clinic with no complaints, indicating a smooth post-operative recovery.

## 3. Discussion

GERD occurs mainly due to the reflux of gastric contents into the esophagus, causing esophageal injury [12]. It is a chronic condition that affects around 15 to 25% of the worldwide population, up to 33.1% of people in the Middle East, and about 45.4% of inhabitants in the Kingdom of Saudi Arabia. It is known that GERD management is a must because leaving it without treatment causes undesirable complications such as impaired quality of life and Barrett’s esophagus which leads to esophageal cancer [1,2,3,4,13,14,15]. The first line of treatment for GERD is Proton Pump Inhibitors, yet, in 20–40% of cases, it leads to unsatisfactory or incomplete responses [5]. As a result, over the past years, there have been some breakthroughs in the field of GERD treatment. This includes laparoscopic and endoscopic advances. Surgical fundoplication has been considered the gold standard procedure for treating GERD. However, its use has been declining in recent years, particularly between the years 2009 and 2013 [6,16]. The decline was associated with an increase in the use of endoscopic techniques, which are less invasive and are said to be associated with less post-operative bloating and dysphagia compared to surgical fundoplication, hence making it appealing to some patients [7,17].

When the EF was introduced in 2006, it faced a lot of doubtful opinions from practitioners managing GERD. Later, data on safety and efficacy were published, which allowed this approach to evolve and is now “associated with solid clinical outcomes, reliably demonstrated patient benefit, and a remarkable safety profile” [8]. Yet, this procedure, like any other, presents some adverse events such as perforation (19.8%), lacerations 17.6%, bleeding (9.2%), and pleural effusions (9.2%). These complications were mostly treated using endoscopic clips (12.3%), chest tube/drain insertion (12.3%), endoscopic retriever devices (11.1%), esophageal stents (8.6%), and surgery (11.1%) [4]. Interestingly, to date, none of the papers published on this technique mentioned a similar condition (Gastrointestinal-Gastric Fistula post EF) to the case presented by the authors in this article. So, to the authors’ knowledge, this is the first case of its kind to be reported in literature. Upon searching the literature, we came across a single case report describing an Esophageo-pulmonary fistula following an EF. The patient presented with a lung abscess caused by an esophageal leak, which required surgical management by a thoracotomy approach [18,19,20].

The case presented here is a gastro-gastric fistula formation from EF, which is a rare complication of endoscopic fundoplication that requires surgical intervention and repair with a redo of the fundoplication. We do not have enough information about the details of the endoscopic procedure as it was performed in another country, and the patient did not present detailed data. Yet, he reported being managed for hemothorax of the right chest following the EF. He required ICU admission and a chest tube insertion. The authors assume that this condition was aggravated by post-procedure bleeding, a hematoma at the suture site, encouraging infection, and, as a result, the later formation of this fistula. The location of the fistula was at the stitch insertion site of the endoscopic suturing device used for fundoplication, causing the failure of the procedure and the presented complication. As a result, he reported recurrent GERD and even worsening of original symptoms.

Furthermore, the authors point out that such cases can be misdiagnosed using imaging and endoscopy, similar to what happened to the patient in the described case. In literature, a meta-analysis by Menon et al. Mentioned that about 11.3% of upper gastrointestinal lesions were overlooked on the upper GI endoscopy [21]. Likewise, in this case, the pre-operative upper endoscopy did not pick up the presence of a fistula; hence, it was an intra-operative diagnosis.

We emphasize keeping this complication in mind when dealing with such cases, putting in mind that even by endoscopy, the fistula could be missed as it is difficult to diagnose, and the only way of diagnosing it could be intraoperatively, as happened in this case. In addition, the authors recommend anticipating and putting this complication in mind, planning for possible management options ahead in order to be able to manage surprises efficiently.

## 4. Conclusions

Gastro-gastric fistula formation following endoscopic fundoplication is a rare complication, and to the best of our knowledge, it has never been reported in the literature. We are reporting this case in order to provide the reader with an insight into one of the possible outcomes of endoscopic fundoplication.

Keeping it in mind as one of the differential diagnoses is important in order to plan surgical management.

Such cases will present you with reflux and intolerance to oral intake after EF, which will prompt meticulous workup and treatment. After all, they may often be missed on imaging and endoscopy as it is hard to pick up the tract due to fibrosis.

The case draws attention to the possible long-term complications associated with EF, even though it is considered to be minimally invasive; however, it can lead to severe consequences years after the procedure. This highlights the need for healthcare providers to be extra vigilant in the early identification of late EF complications, as demonstrated in this report.

The authors of this paper emphasize that failed endoscopic cases should be best treated by a surgical approach in order to recognize and manage such conditions safely.

In light of the reported case, we recommend that healthcare professionals involved in the management of GERD be vigilant about the possible late complications and adverse events of EF. There is also a need for Randomized Control Trials (RCTs) and longitudinal studies to foster a better understanding of the risk factors and incidences of late complications of EF, which could improve the development of evidence-based management and strategies for GERD patients.

## Data Availability

The data underlying this study are available in this article.

## References

[1] Fry L.C., Mönkemüller K., Malfertheiner P. (2007). Systematic review: Endoluminal therapy for gastro-oesophageal reflux disease: Evidence from clinical trials. Eur. J. Gastroenterol. Hepatol..

[2] Madan A.K., Ternovits C.A., Tichansky D.S. (2006). Emerging endoluminal therapies for gastroesophageal reflux disease: Adverse events. Am. J. Surg..

[3] Brar T.S., Draganov P.V., Yang D. (2017). Endoluminal Therapy for Gastroesophageal Reflux Disease: In Between the Pill and the Knife?. Dig. Dis. Sci..

[4] Fernando H.C. (2017). Endoscopic fundoplication: Patient selection and technique. J. Vis. Surg..

[5] Sobrino-Cossío S., Soto-Pérez J., Coss-Adame E., Mateos-Pérez G., Matsubara O.T., Tawil J., Vallejo-Soto M., Sáez-Ríos A., Vargas-Romero J., Zárate-Guzmán A. (2017). Post-fundoplication symptoms and complications: Diagnostic approach and treatment. Rev. Gastroenterol. México.

[6] Chen S., Du F., Zhong C., Liu C., Wang X., Chen Y., Wang G., Gao X., Zhang L., Li L. (2021). Gastroesophageal reflux disease: Recent innovations in endoscopic assessment and treatment. Gastroenterol. Rep..

[7] de Santiago E.R., Albéniz E., Estremera-Arevalo F., Sanchez-Vegazo C.T., Lorenzo-Zúñiga V. (2021). Endoscopic anti-reflux therapy for gastroesophageal reflux disease. World J. Gastroenterol..

[8] Testoni P.A., Mazzoleni G., Testoni S.G.G. (2016). Transoral incisionless fundoplication for gastro-esophageal reflux disease: Techniques and outcomes. World J. Gastrointest. Pharmacol. Ther..

[9] Frazzoni M., Piccoli M., Conigliaro R., Frazzoni L., Melotti G. (2014). Laparoscopic fundoplication for gastroesophageal reflux disease. World J. Gastroenterol..

[10] Huang X., Chen S., Zhao H., Zeng X., Lian J., Tseng Y., Chen J. (2017). Efficacy of transoral incisionless fundo- plication (TIF) for the treatment of GERD: A systematic review with meta-analysis. Surg. Endosc..

[11] Kempen J.H. (2011). Appropriate Use and Reporting of Uncontrolled Case Series in the Medical Literature. Am. J. Ophthalmol..

[12] Witteman B.P.L., Conchillo J.M., Rinsma N.F., Betzel B., Peeters A., Koek G.H., Stassen L.P.S., Bouvy N.D. (2015). Randomized controlled trial of transoral incisionless fundoplication vs. proton pump inhibitors for treatment of gastroesophageal reflux disease. Off. J. Am. Coll. Gastroenterol..

[13] Sfara A., Dumitrașcu D.L. (2019). The management of hiatal hernia: An update on diagnosis and treatment. Med. Pharm. Rep..

[14] Kahrilas P.J. (2003). GERD pathogenesis, pathophysiology, and clinical manifestations. Clin. J. Med..

[15] Alsuwat O.B., Alzahrani A.A., Alzhrani M.A., Alkhathami A.M., Mahfouz M.E.M. (2018). Prevalence of gastroesophageal reflux disease in Saudi Arabia. J. Clin. Med. Res..

[16] Kellerman R., Kintanar T. (2017). Gastroesophageal reflux disease. Prim. Care.

[17] Zaterka S., Marion S.B., Roveda F., Perrotti M.A., Chinzon D. (2019). Historical perspective of gastroesophageal reflux disease clinical treatment. Arq. Gastroenterol..

[18] Richardson W.S., Gorham J.K., Neal N., Fanelli R.D. (2022). Endoscopic Treatment of Gastroesophageal Reflux Disease. Adv. Surg..

[19] Ramai D., Shapiro A., Barakat M., Facciorusso A., Dull A., Chandan S., Adler D.G. (2022). Adverse events associated with transoral incisionless fundoplication (TIF) for chronic gastroesophageal reflux disease: A MAUDE database analysis. Surg. Endosc..

[20] Titus J.M., Mason D.P., Raymond D.P., Rice T.W., Murthy S.C. (2013). Esophagopulmonary fistula and left lung abscess after transoral inci-sionless fundoplication. Ann. Thorac. Surg..

[21] Menon S., Trudgill N. (2014). How commonly is upper gastrointestinal cancer missed at endoscopy?. Endosc. Int. Open.

